# Pulvinar thalamic nucleus allows for asynchronous spike propagation through the cortex

**DOI:** 10.3389/fncom.2015.00060

**Published:** 2015-05-19

**Authors:** Nelson Cortes, Carl van Vreeswijk

**Affiliations:** ^1^Institut de la Vision, UMRS 968 UPMC, INSERM, Centre National de la Recherche Scientifique U7210, CHNO Quinze-VingtsParis, France; ^2^Physiology and Pathology, Center for Neurophysics, University Paris Descartes and Centre National de la Recherche Scientifique UMR 8119Paris, France

**Keywords:** feedforward network, cortical transmission, pulvinar nucleus of the thalamus, cortico-thalamo-cortical connections, asynchronous spike transmission, balanced network

## Abstract

We create two multilayered feedforward networks composed of excitatory and inhibitory integrate-and-fire neurons in the balanced state to investigate the role of cortico-pulvino-cortical connections. The first network consists of ten feedforward levels where a Poisson spike train with varying firing rate is applied as an input in layer one. Although the balanced state partially avoids spike synchronization during the transmission, the average firing-rate in the last layer either decays or saturates depending on the feedforward pathway gain. The last layer activity is almost independent of the input even for a carefully chosen intermediate gain. Adding connections to the feedforward pathway by a nine areas Pulvinar structure improves the firing-rate propagation to become almost linear among layers. Incoming strong pulvinar spikes balance the low feedforward gain to have a unit input-output relation in the last layer. Pulvinar neurons evoke a bimodal activity depending on the magnitude input: synchronized spike bursts between 20 and 80 Hz and an asynchronous activity for very both low and high frequency inputs. In the first regime, spikes of last feedforward layer neurons are asynchronous with weak, low frequency, oscillations in the rate. Here, the uncorrelated incoming feedforward pathway washes out the synchronized thalamic bursts. In the second regime, spikes in the whole network are asynchronous. As the number of cortical layers increases, long-range pulvinar connections can link directly two or more cortical stages avoiding their either saturation or gradual activity falling. The Pulvinar acts as a shortcut that supplies the input-output firing-rate relationship of two separated cortical areas without changing the strength of connections in the feedforward pathway.

## 1. Introduction

In the cortex, information about sensory stimuli has to be transmitted through several areas to extract different aspects of the stimulus. In primates, this is a particularly hard task with visual information because the visual hierarchy has at least 10 levels (Felleman and Van Essen, 1991). We have recently investigated the question how this can be achieved using a rate based model where the arrangement of the connections was hierarchical: the activity from the previous layer is integrated and then non-linearly transmitted to the next level (Cortes and van Vreeswijk, 2012). The results of this study show that, while cortico-cortical connections are devoted to the structure and creation of visual receptive fields through hierarchical levels,the network shows a poor representation of the stimulus contrast in the absence of an interaction with a thalamic structure. In contrast, when the cortical network is connected to a network representing the pulvinar nucleus of the thalamus, information about the stimulus contrast is present in all levels of the hierarchy. Additionally,t he tuning properties of the receptive fields are close to contrast invariant throughout the hierarchy.

However, rate models assume that spike synchrony can be neglected and it is not clear if this is the case. Build up of synchrony synchrony could easily occur as the activity flows through the hierarchy, and this might disrupt the computational capacity of the system. Here we therefore study how information about the input can be transmitted through a feedforward network (FFN) of *spiking*. We consider a simple model which consists of a chain of layers of excitatory (E) and inhibitory (I) neurons in which a neuron in a given layer receives multiple synaptic inputs from E neurons in the previous layer and E neurons send out projections to the next layer. Within the layer the E and I neurons are sparsely coupled.

Two types of information can be carried through the chain in such a model: the firing rate of neurons or a temporal pattern of activity (van Rossum et al., 2002; Litvak et al., 2003; Vogels and Abbott, 2005; Kumar et al., 2010). In a “rate code” paradigm, the neurons fire roughly asynchronously and the average firing rate in a previous layer determines the activity of the next, generating an output rate that is related uniquely to the input (Wilson and Cowan, 1972; Kistler and Gerstner, 2002; Kumar et al., 2008). In the “temporal code,” information is carried by groups of neurons that fire with almost no delay between them (Diesmann et al., 1999). Previous works have shown that rate and temporal code can not coexist and a network that has a synchrony pattern of transmission will flip to an asynchronous activity, and vice versa (Mehring et al., 2003; Kumar et al., 2010). As we will show in our results, as for those previous network architectures propagating either rate or temporal spike activity, if a FFN has many layers, the last layer always has an input-output curve either with a small slope, a step shape, or saturated behavior (Cortes and van Vreeswijk, 2012).

Therefore, in this study we present an alternative way to transmit information about the stimulus in such a chain. This is done by allowing for a second pathway to transmit this information that shortcuts the flow of information through the levels. To propagate variation of the input rate through the chain, our visual cortex, we create an external structure that simulates the Pulvinar nucleus of the thalamus (Pul). The Pul is the good candidate because its particular arrangement of connectivity with the visual cortex (Shipp, 2001, 2003; Kaas and Lyon, 2007; Sherman, 2007), and as pulvinar activation/suppression modifies temporal (Molotchnikoff and Shumikhina, 1996; Shumikhina and Molotchnikoff, 1999) or spatial (Soares et al., 2004) properties of visual cortical RFs. In FFN the main challenge is to adjust the gain of transmission to get a proper linear transmission between layers. Pul, given its shortcut property, supplies the gain in the transmission of cortical spikes which allows an almost linear firing rate transmission throughout the cortex. Because of the thalamic pathway, the gain transmission through the feedforward pathway can be lower and this prevents the build up of spike correlations in the chain.

## 2. Materials and methods

### 2.1 Cortical neurons

The *N_A_* neurons of population *A* = *E, I* in each layer of the cortical chain are modeled as linear integrate-and-fired (IF) neurons with equal membrane time constant, τ*_m_* ≡ *C_M_*/*g_L_* = 10 ms, and “resting membrane potential,” *v_res_*. Here, *C_M_* and *g_L_* are the membrane capacitance and the leak conductance, respectively. The sub-threshold dynamics of the membrane potential, *v*^*A*,ℓ^_*i*_ of neuron *i* of population *A* in layer ℓ, satisfies

(1) $$C_{M}\frac{dv_{i}^{A,\ell }}{dt}=-g_{L}(v_{i}^{A,\ell }-v_{res})+i_{i}^{A,\ell }(t),$$

where *i*^*A*,ℓ^_*i*_(*t*) is the total synaptic current into the neuron. If the membrane potential reaches the spike threshold *v_thr_* at time *t*, a spike is emitted, and the voltage reset at time *t* + τ*_ref_* to the reset potential, *v_reset_*, which we assume to be equal to the resting potential, *v_reset_* = *v_res_*. For the refractory period we use, τ*_ref_* = 5 ms.

We can rescale the membrane potential to a dimensionless variable, *v*^*A*,ℓ^_*i*_ = (*v*^*A*,ℓ^_*i*_ − *v_res_*)/(*v_thr_* − *v_res_*). In this rescaled variables the threshold satisfies *v_thr_* = 1, while for the resting potential we have *v_res_* = 0. For *v*^*A*,ℓ^_*i*_ we can write

(2) $$\tau_{m}\frac{dV_{i}^{A,\ell }}{dt}=-V_{i}^{A,\ell }+I_{i}^{A,\ell }(t),$$

where *I*^*A*,ℓ^_*i*_ = *i*^*A*,ℓ^_*i*_/[*g_L_*(*v_thr_* − *v_res_*].

### 2.2 Feedforward network

We create a feedforward network (FFN) of *L* = 10 layers in which each layer has equal number of excitatory and inhibitory IF neurons, *N_E_* = *N_I_* = 3000. In the FFN, activities of neurons in a layer is due to input from the excitatory neurons in the previous layer, except for layer 1 which receives activity from an external source. *E* neurons from layer ℓ are connected randomly to both *E* and *I* neurons in ℓ + 1 with a connection probability of 10%. There are also reciprocal inputs, which originate from the *E* and *I* neurons in the same layer. The reciprocal connections are also random with a probability of connection of *p* = 0.1. Thus, on average, each neuron receives *K* inputs from the *E* cells in the previous layer, and from *K_E_* and *K_I_* neurons in its own layer, where *K* = 300. In the FFN the input current *I*^*A*,*l*^_*i*_(*t*) can be written as

(3) $$I_{i}^{A,\ell }(t)=I_{rec,i}^{A,\ell }(t)+I_{F\;F,i}^{A,\ell }(t),$$

where the recurrent input, *I*^*A*,ℓ^_*rec,i*_(*t*), is given by

(4) $$I_{rec,i}^{A,\ell }(t)=\sum_{B\;=\;E,I}J_{ij}^{AB,\ell }E_{j}^{B,\ell }(t),$$

while, the feedforward input, *I*^*A*,ℓ^_*FF*_(*t*), satisfies

(5) $$I_{F\;F}^{A,\ell }(t)=\sum_{j}J_{ij}^{A0,\ell }E_{j}^{E,\ell -1}.$$

Here the coupling matrices *J*^*AB*,ℓ^_*ij*_ for *A* = *E, I* and *B* = *E*, *I*, 0 are random matrices with *J*^*AB*,ℓ^_*ij*_ =*J_AB_* with probability *p* and *J^AB,ℓ^_ij_* =0 otherwise. *J_AB_* is positive if the presynaptic neurons is excitatory, *J_AB_* > 0 for *B* = *E*,0 and negative for inhibitory presynaptic cells, *J_AI_* < 0.

For ℓ ≥ 1, *E*^*A*,*l*^_*j*_ is the activation of the synapse of neuron *i* of population *A* in layer ℓ. This is given by

(6) $$E_{j}^{A,\ell }(t)=\sum_{t_{j(k)}^{A,t}<t}\epsilon_{A}(t-t_{j(k)}^{A,\ell }),$$

where *t*^*A*,*t*^_*j*(*k*)_ is the time of the *K*th spike on neuron *j* of population *A* and layer ℓ, and ϵ*_A_* is the scaled response to a single spike, which is described by an α- function

(7) $$\epsilon_{A}(t)=\frac{t}{{\left[\tau_{syn}^{B}\right]}^{2}}e^{-t/\tau_{syn}^{B}},$$

for *t* ≥ 0, otherwise ϵ*_A_* = 0. It has been previously shown that networks with such strong coupling can evolve to the balance stated if the excitatory feedback is sufficiently slow relative to the inhibitory one (van Vreeswijk and Sompolinsky, 1998). Otherwise they produce strong synchronous activity. To avoid this problem we assume that the excitatory and inhibitory synaptic time constants are given by τ*^E^* = 4 ms and τ^*I* = 1^ ms, respectively.

For ℓ = 0, *E*^*E*,0^_*i*_ gives the synaptic activation of the LGN cells which project to the first layer. We do not model these cells explicitly, but assume that they follow Poisson statistics. Thus, for each *i* we independently generate a Poisson spike train *t^E0^*_*i*,(*k*)_ with a rate *r^0^*(*t*). The synaptic activation, *E*^*E*,0^_*i*_ is then given by Equation (6).

### 2.3 Pulvinar-feedforward network

In the Pulvinar-Feedforward network we have a cortical FFN as described above interacting with a pulvinar like structure. We create this system taking into account three special properties of the Pul. (i) Connections from the Cortex/Pul to the Pul/Cortex have a gradient from lower to higher hierarchical cortical levels (Shipp, 2001, 2003). In the model, low Pul sub-units are connected with low cortical areas, higher cortical areas with higher Pul sub-units. (ii) The cortico-thalamo-cortical loop is open (Guillery and Sherman, 2002; Sherman, 2007). In he model there is a non-reciprocal connectivity between cortex and Pul, neurons from cortical area ℓ project to neurons in sub-unit ℓ Pul. These in turn project to cortical ℓ +1. (iii) Besides the local connectivity, there are non-local connections in the Pul circuitry, mediated by long range inhibitory interneurons (Imura and Rockland, 2006, 2007). These connections are implemented as inhibitory connections among Pul sub-units.

We create a Pul network which consists of *L* − 1 sub-units. Each sub-unit consists of 600 *E* and 600 *I* neurons. The neurons in the Pul are also modeled as linear integrate and fire neurons. The voltage *v*^*A*,ℓ^_*pul*,*i*_ of pulvinar unit *i* of population *A* in layer ℓ satisfies Equation (1), where its synaptic input, *I*^*A*,ℓ^_*pul*,*i*_ consists of 4 terms

(8) $$I_{pul,i}^{A,\ell }(t)=I_{rec,i}^{A,\ell }(t)+I_{P\text{​}P,i}^{A,\ell }(t)+I_{PC,i}^{A,\ell }(t)+I_{LR,i}^{A,\ell }(t),$$

where *I^A,ℓ^_rec,i_* is the recurrent input from the sub-unit, *I^A,ℓ^_PP,i_* is feedforward input from the previous sub-unit, *I^A,ℓ^_LR,i_* is the the input from long-range inhibitory neurons from elsewhere in the Pul and *I^A,ℓ^_PC,i_* is the input from the cortex.

Both recurrent and feedforward inputs are similar to those from the FFN, so they are described by Equations (4) and (5), respectively. Here we have to replace *E^A,ℓ^_i_* by *E^A,ℓ^_pul,i_* the synaptic activity of pulvinar neuron *i* in population *A* of layer ℓ and *J^AB^_ij_* should be replaced by the connection matrix of the Pul, *J^AB^_pul,ij_*, for which we have *J^AB^_pul,ij_* = *J^pul^_AB_* with a probability *p* = 0.5, so that on average the pulvinar neurons receive input from *K* = 300 recurrent excitatory, recurrent inhibitory and feedforward inputs. In the case of the feedforward current, however, the input for the layer 1 in the pulvinar is from layer 1 of the FFN. We use the subscript *PP* to differentiate the pulvinar feedforward input from the feedforward pathway of the FFN.

The excitatory input from FFN to layers ℓ of the pulvinar, *I^A,ℓ^_PC,i_*, is only from *E* neuron layer ℓ of the cortex. It is given by

(9) $$I_{PC,i}^{A,\ell }(t)=\sum_{j}J_{ij}^{AE,\ell }(PC)E_{ctx,j}^{E,\ell }(t),$$

where we now have written *E^E,ℓ^_ctx,j_* for *E^E,ℓ^_j_* to avoid confusion. The random connection matrix *J^AE,ℓ^_ij_(PC)* satisfies *J^AE,ℓ^_ij_*(*PC*) = *J^PC^_AE_*/$\sqrt{K}$ with probability 0.1 and 0 otherwise.

The current from the long-range connections in neurons of layer ℓ^0^ of the pulvinar, *I*^*A*,ℓ_0_^_*LR*,*i*_(*t*), consists of inhibitory neurons from layers ℓ ≠ ℓ_0_, and is given by

(10) $$I_{LR,i}^{A,\ell_{0}}(t)=\sum_{\ell \;=\;1,\ell_{0}\neq \ell }^{L-1}\sum_{j}J_{ij}^{AI,\ell_{0}}(LR)E_{pul,j}^{I,\ell_{0}}(t),$$

where *J*^*AI*,ℓ_0_^_*ij*_(*LR*) = *J^LR^_AI_*/$\sqrt{K}$ with probability 1/[2(*L*−2)] and zero otherwise.

Finally, to complete the cortico-thalamic-cortical loop, we inject the input from *E* neurons in pulvinar layer ℓ − 1 into excitatory input into the FFN in layer *ℓ, I*^*A*, ℓ^_*CP,i*_(*t*), which is given by

(11) $$I_{CP,i}^{A,\ell }(t)=\sum_{j}J_{ij}^{AE,\ell }(CP)E_{pul,j}^{E,\ell -1}(t),$$

while *I*^*A*,1^_*CP*,*i*_(*t*) = 0 and *J*^*AE*,ℓ^_*ij*_(*CP*) = *J^CP^_AE_*/$\sqrt{K}$ with probability 0.5.

As a final remark: when we are dealing with the cortico-pulvinar network we denote the synaptic connection within the cortex by *J^Ctx^_A_* rather than *J_AB_* to avoid confusion.

**Figure 1:**
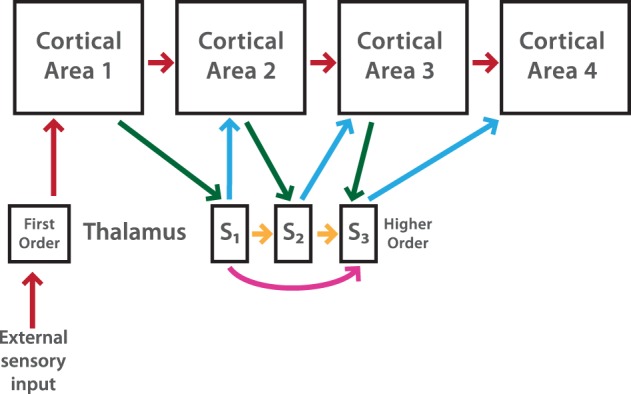
**Schematic diagrams showing organizational features of cortico-thalamic-cortical (CTC) loops**. First order (FO) nuclei (e.g., LGN) receive drive input from sensory cells. A FO nucleus represents the first relay from the thalamus to the primary cortical area. The CTC pathway includes the cortico-cortical (CC) pathway, in this case only feedforward connections (red arrows), but it also takes into account non-reciprocal connections from a higher order (HO) thalamic nucleus (e.g., the Pulvinar). To create a non-reciprocal loop between cortex and thalamus, cortical neurons in layer ℓ receive input from thalamic subdivision ℓ−1, while this subdivision sends input to neurons in cortical area ℓ. The transthalamic pathway (green and blue arrows) assumes that the activity flow passes from one cortical area to another area through the HO thalamic nucleus. This is possible by long-range connections inside the thalamus (magenta arrows). For simplicity only one thalamic subdivision (*S_3_*) shows these connections. Thalamic HO neurons in subdivision ℓ receive inputs from neurons in subdivision ℓ −1 and also from neurons in subdivisions 1, 2,…, ℓ − 2. A feedforward pathway through thalamic subdivisions is also considered (orange arrows). These connections are assumed in our cortex-thalamus network. Redrawn after Hegde and Felleman (2007).

Figure 1 shows a schematic representation of the cortex-thalamus network which takes into account the previously described connections.

### 2.4 Optimization criteria

We optimize the input-output response of the last cortical layer, the relation between the firing rate of the last layer and the rate of the input applied to the first layer, to summarize the performance of the cortico-pulvinar network. Optimal transmission is reflected by a curve which varies linearly with the input, *r*^0^, and spans the dynamic range of outputs maximally. As optimization criterion we use the entropy of the output rate distribution hen the input rate *r*_0_ is distributed uniformly between 10 and 100 Hz. This entropy is low when both the input-output relation is close to a step function and when the dynamic range is small. Thus, to optimize the output of unit *L* of the cortico-pulvinar network when a homogeneous input is applied in layer 1, we determine parameters for which the entropy, *H*, given by

(12) $$H=-\int_{10}^{100}drP_{L}(r)\log P_{L}(r)$$

is maximal. Here, *P_L_*(*r*) is the probability density distribution of the *L*th layer where *L* = 10.

We only consider the optimal input-output distribution of the large layer, because we found that optimizing this automatically also leads to a nearly linear input-output relation wit a large dynamic range for intermediate layers.

We now derive an expression for *H*, where the relation *r_L_* = *F*(*r*_0_) is known and *r*_0_ is drawn from a homogeneous distribution between 10 and 100 Hz. For a small Δ*r* this probability will be

(13) $$P_{L}(r)\Delta r=\text{Prob}(r<r_{L}<r+\Delta r).$$

Since *r_L_* = *F*(*r_0_*), this is equal to

(14) $$\begin{array}{l} P_{L}(r)\Delta r=\text{Prob}\left(F^{-1}(r)<r_{0}<F^{-1}(r+\Delta r)\right)\\ \;\approx \text{Prob}(F^{-1}(r)\text{ ​}<\text{​}r_{0}\text{​ }<\text{​}F^{-1}(r)\text{​}+\text{​}s{F^{\prime }}^{-1}(r)\Delta r). \end{array}$$

Here *F*'^−1^ is the derivative of *F*^−1^, the inverse of *F*. Since *r*_0_ is drawn from a uniform distribution between 10 and 100 Hz, Prob(*F*^−1^(*r*) < r_0_ < *F*^−1^ (*r*) + *F*'^−1^ (*r*) Δ *r*) is equal to *F*'^−1^ (*r*) Δ*r*. Together with *F*'^−1^ (*r_L_*) = 1/*F*' (*r*_0_) this yields

(15) $$P_{L}(r_{L})=\frac{1}{F^{\prime }(r_{0})}.$$

Inserting this into Equation (12), the entropy is given by

(16) $$H=\int_{10}^{100}dr_{0}\log F^{\prime }(r_{0}),$$

where we have used *dr_L_* = *F*'(*r*_0_)*dr*_0_. We numerically optimize the nine parameters of the cortico-pulvinar network by using the Powell's method in multiple dimensions (Press et al., 1992).

## 3. Results

Our goal is to achieve a coherent transmission of firing rate through two different network architectures: a purely FFN and a FFN coupled with a parallel small network. A coherent propagation has to preserve the frequency input injected in the first layer as much as possible, and so, a last layer having a large representation of outputs. The input-output response of the last layer should then be similar to the neuronal responses of visual cortical areas to contrast input variations (Sclar et al., 1990; Avidan et al., 2002). Before we study how activity propagates in such networks we first analyze the input-output response of a single layer, i.e., we consider a network with *L* = 1. We explore the conditions to obtain a linear input-output response in which its slope, the gain, is a unit. We will then describe the response of a chain of several layers connected feedforwardly. We will show that it is challenging to maintain a coherent transmission through a purely FFN because the firing rate either is tiny represented in the last layer or synchronized-spikes break the linearity of the propagation across layers. Finally, we will demonstrate that a FFN coupled to a pulvinar-like structure can dramatically improve the linear transmission of the input rate solving the problem which has the purely feedforward architecture.

### 3.1 Input-output relations for a network in the balanced state

We simulated a layer of 6000 neurons with equal number of excitatory (*E*) and inhibitory (*I*) cells to obtain a network with balanced state properties. Each population of each layer receives 300 uncorrelated excitatory Poisson spikes while the average rate of the external input varied in frequency between 10 and 100 Hz. Also, recurrent connectivity inside each layer is implemented where neurons of each population receives input from, on average, 300 excitatory and 300 inhibitory neurons chosen at random (see Section 2). Neurons receive, on average input from *K* = 300 neurons in the respective populations, while the strength of the inputs, relative to the threshold input, is of order 1/$\sqrt{K}$. Such strong connections induces the fact that the network evolves to state in which the total excitatory input is approximately balanced by the total inhibitory input if *K* is large, *J_E0_*/*J_I0_* > *J_EI_*/*J_II_* > *J_EE_*/*J_IE_* and the excitatory synaptic time constant is sufficiently larger than the inhibitory (van Vreeswijk and Sompolinsky, 1996, 1998). Under this regime, the activity of the neurons changes only very weakly if one rescales all the synaptic efficacies of a given population by the same factor. Thus, in networks used throughout this study the strength of the feedforward and the recurrent both excitatory (*E*) and inhibitory (*I*) connections to populations *A* = *E, I* are scaled as *J_A0_*/$\sqrt{K}$, *J_AE_*/$\sqrt{K}$, and *J_AI_*/$\sqrt{K}$, respectively, and we further reduce the other of parameters by using *J_EE_* = *J_IE_* > 1.

The balanced state is characterized by irregular activity of the neurons with a substantial heterogeneity of the rates, while in the large *K* limit the average rates of the excitatory and inhibitory neurons scale linearly to the rate of the external input. In the large *K* limit it is rather straightforward to get that the excitatory population firing rate is the same as the external input rate which is simply a matter of choosing the synaptic weights *J_AB_* appropriately. However, for finite *K* the deviations from this solution are rather severe. Since we use a small *K* (*K* = 300), we can expect a substantial deviation from this solution. Thus, the question is whether in our network we can choose parameters *J_AB_* such that the output of the network tracks the input if the input rate varies between 10 and 100 Hz.

**Figure 2:**
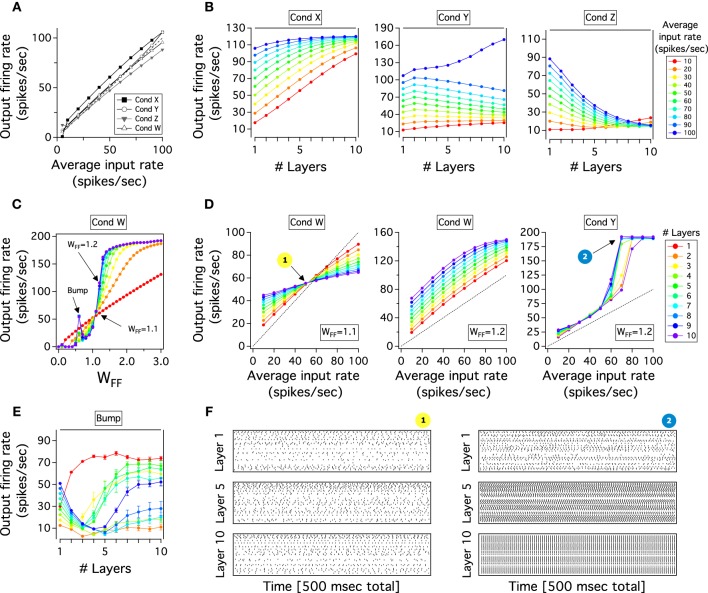
**Performance of single and FFN with reciprocal *E* excitatory and *i* inhibitory neurons in the balanced state. (A)** Representative input-output curves of the excitatory population for 4 different conditions (Cond). In Cond X the gain is larger than 1 and *J_AE_* < *J_II_*, Cond Y the gain is larger than 1 and *J_AE_*≈ *J_II_*, Cond Z the gain is smaller than 1 and *J_AE_* ≈ *J_II_*, Cond W the gain is almost a unit and *J_AE_* > *J_II_*. **(B)** Output firing rate as a function of layers for a FFN which receives different average input rates (right color scale from 10 to 100 Hz). Parameter values from conditions of the single layer as **(A)**. **(C)** Output firing rates of feedforward layers for a constant input (50 Hz) when **W*_FF_* increases from 0 to 3. Layers from 1 to 10 are represented in colors as scale in **(D)**. **(D)** Input-output curves for feedforward layers (right color scale) of networks at different conditions. Parameter conditions for each network as **(A)**. The transmission of firing rate strongly depend of the first layer gain. **(E)** Transmission through a FFN for the bump condition in **(C)**. Color scale for layers as **(D)**. **(F)** Raster plots show emergence of synchronized activity for points indicated in **(D)**. 100 random chosen excitatory spikes of layer 1, 5, and 10 are plotted as a function of 500 ms. For all figures, except for **(F)**, each curve is an average result of 50 independent simulations. For more details see text.

Our simulations show that it is rather difficult to find values for an exactly linear input-output relation with a 1:1 ratio when a small network receives an input which varies gradually in firing rate. The input-output curves show a small nonlinearity for either low or high frequency inputs. In fact, a trade-off of a good performance between low and high input rates need to be made: an optimal almost linear solution is found when synaptic efficacies are relatively large. For instance, the strength from inhibitory to excitatory populations has to be *J_EI_* > $\sqrt{K}$. That is, because the threshold is equal to 1, one inhibitory spike would decrease the voltage by more than the distance between the resting potential and the threshold. Of course if we increase the size of the network and increase *K* the efficacy of the single synapse can be reduced. Later we shall see the implications of this trade-off in the transmission of activity in long chains when we have a small size network. In our networks, the approach used to solve *J_EI_* > $\sqrt{K}$, is to consider a larger membrane time constant, τ*_m_*, increasing the time over which spikes are integrated. Thus, even when having a small size network, our aim to increase or decrease the gain of curves is achieved when both external and inhibitory coupling, *J_A0_*, are fixed and the strength of the excitatory connectivities, *J_AE_*, is gradually changed (Figure 2A).

Figure 2A captures these results. For simplicity we only plot the responses of the excitatory neuronal populations. We show two representative conditions in which the gain is slightly both large (Cond X) and small (Cond Z), and two other cases when the gain is almost a unit (Cond Y and W). To obtain these results the excitatory strengths, *J_AE_* and *J_0E_*, are lower (Cond X), similar to (Cond Z and Y) or higher (Cond W) than *J_II_*. In Cond X and Z the input-output curve shows a small nonlinearity at low or high firing rates with a slope larger or smaller than 1, respectively. Although we found that the gain can also slightly decrease by only increasing the strength of the recurrent excitatory connections in Cond X, for the effects of the feedforward transmission the results are similar (see next section). In Cond Y and W, it can be seen that the nonlinearity of the curves becomes smaller when *J_AE_* and *J_A0_* are large. In fact, the external strength to the network is equal between Cond Z and Cond Y but the latter has stronger excitatory recurrent connections. However, for both conditions the nonlinearity still persists with the gain tiny crossing the equality line. For instance, Cond W is the closest linear output over the whole dynamic range but curves are obtained when *J_AE_* is small: slightly smaller recurrent excitatory weights produce a saturated output activity at higher input rate (not shown). Thus, although we can easily modify the gain of a single layer by changing weights of excitatory compare to inhibitory recurrent connections and keeping necessary conditions to have a balanced state, it is hard to attain an accurate linear gain of 1: the nonlinearities at either higher or lower firing rate outputs produce that the input-output curve crosses the line *x* = *y*. In the next section, we will see how this irregular linearity influences the transmission of activity in a multilayer network.

### 3.2 Transmission of firing rates in a FFN with layers in the balanced state

It has been previously suggested that FFN which layers in the balanced state might produce a linear transmission of firing when the gain is close to a unit (Litvak et al., 2003). Such propagation of input might be similar to transmission of firing rate observed in the visual cortex when a contrast input is varied (Sclar et al., 1990; Avidan et al., 2002) which in primates suggests to cross at least 10 hierarchical cortical levels (Felleman and Van Essen, 1991; Hegde and Felleman, 2007). We will explore whether the transmission of rates in a chain of ten neuronal layers in the balanced state is feasible when each layer has identical populations parameters of a single balanced network, and so, all layers have the same strength of both feedforward and recurrent connectivity. As before, a pool of 300 excitatory Poisson spikes with rate *r*_0_ determines the input which targets with a probability of *p* = 0.1 excitatory and inhibitory populations of the first layer. Each layer has strong recurrent feedback assuring the balanced state. In layer ℓ, the probability of a connection between pairs of cells was *p* = 0.1 just as for the feedforward connections. For ℓ ≥ 2 the neurons receive feedforward input from on average 300 randomly chosen *E* neurons in layer ℓ−1 (see Section 2). Similar network architectures have shown that feedforward transmission with this probability of connection produced low levels of synchrony in the chain (Shadlen and Newsome, 1998; Litvak et al., 2003).

We tested the transmission of firing rate when a small, large and almost unit gain is imposed to the multilayer network. As it can be seen in Figure 2B, the transmission of firing rate strongly depends on the gain of transmission of the first layer. In Cond X, when the gain is larger than 1 and *J_AE_* < *J_II_*, the transmission of firing rate inputs from 10 to 100 Hz, *r*_0_, progressively increases as a function of layers with an activity partially saturating into a single firing rate output (~110 Hz) at deep feedforward layers. In Cond Z, when the gain is lower than 1 and *J_AE_* = *J_II_*, firing rate outputs decay and reach a low fixed activity (~10 Hz) for any input varied in frequency. Both previous networks tend to converge to a single firing rate which is the point where the gain of the first layer crosses the linear *x* = *y* curve. The propagation of firing rate in Cond Y is different to previous networks with either large or small gain. Theoretically, a FFN with many layers, a linear response with a gain of 1 is conducive to a stable feedforward propagation (Cortes and van Vreeswijk, 2012). However, despite the fact that the input-output gain curve of the first layer is almost linear, with *J_AE_* > *J_II_*, the chain is unable to transmit the activity of the external input: firing rates move progressively to slightly lower rates as one approaches the last layer but for high input rate its output bifurcates and goes quickly to its almost maximal rate (1/τ*_ref_*). In this case, if one plots the activity of the last layer against the input rate, one obtains a step response which is unnatural neuronal transmission (Figure 2D, Cond Y). Thus, for Cond X and Z, if the gain is different from 1 the propagation of an input through layers will rapidly diverge to a linear output in the last layer with either an extremely larger, if the gain in the first layer is larger than 1, or an extremely small gain, if the gain is less or equal to 1 in the first layer (Cortes and van Vreeswijk, 2012). This behavior of the FFN is observed when *J_AE_* is small. Similar behavior would be expected when *J_AE_* is larger (Cond Y). However, as we will analyze later, the chain develops synchrony and the average rate evolves quickly to saturation.

#### 3.2.1 Increasing the strength of the feedforward connections

Before analyzing the regimes in which the FFN develops synchrony across layers, we will explore whether changes in the strength of the feedforward pathway solves the problem of a linear transmission. For a given *J_AE_*, the increment of *J_A0_* can produce a near-linear input-output response for the firing rate of the last layer and a faithful rate propagation across layers might be possible (Vogels and Abbott, 2005). As before, we explore the transmission of a signal that varies in firing rate, but in this case it will be only explored for *r*_0_ = 50 Hz.

We study the network response as the *J_A0_* is gradually increased when a constant firing rate is applied to layer 1. *J_A0_* is adjusted by a factor *W_FF_* that symmetrically multiplies excitatory feedforward strengths, *J_E0_* and *J_I0_*. Because *J_A0_* is the same for all layers, this factor enhances all the feedforward transmission. We use the same parameter values as those in Figures 2A,B. Figure 2C shows the average output firing rate in layers as a function of *W_FF_* (Rainbow legend in Figure 2D indicates layers). In general, it is observed that firing rate of *E* populations in all layers shows an increase with increasing *W_FF_*. The activity goes smoothly from low to high firing rates while curves asymptotically saturate when *W_FF_* is large. This gradual transition is steeper as *J_AE_* becomes larger because synchronous spikes generate in the chain and the firing rate for higher layers is almost always in the saturated state (not shown). The plot also shows a point where all firing rates overlap (~60 Hz). In this intersection, all the layers have approximately the same output firing rate to the same *r*_0_ = 50 Hz. As *J_AE_* increases, the crossing output rate moves gradually to high *W_FF_* (not shown). We can also easily distinguish a sudden transition of firing rates in between *W_FF_* = 0 and the intersection point: a “bump” appears through the network. Activity of the layers drops until layer 3-4 to then, for deep layers, rise and reach a firing rate close to that of the last layer. This peak becomes larger as the *J_AE_* increases (not shown). We will further analyze this response of the system in the next section. Thus, despite the “bump,” activities of early layers in the network remain close to that from layer 1, whereas for layers >5 their firing rates reach quickly similar responses of the last layer as *W_FF_* gradually increases.

We will now explore the transmission when the firing rate *r*_0_ is varied in frequency (10–100 Hz) and plot the input-output response of layers through the chain to examine whether the variation of *W_FF_* can produce a faithful transmission of firing through the chain of layers. If this is true, the last layer should maintain a close to linear input-output relation with a large dynamic range. We will investigate the two representative *W_FF_* indicated as arrows in the previous plot (Figure 2C). For reference, we take two representative strengths: where the firing rate of layers overlaps and another after this point. We ignore *W_FF_* before the firing rate intersection because propagation of firing rates across the FFN is similar to that in Cond Z of Figure 2B, in which the input-output response of layer will gradually decrease and the final layer will have an almost flat gain. This is a comparable behavior for *W_FF_* = 1.1. If as one moves from low to higher layers, as Figure 2C shows, curves become flat in slope and, for the last layer, the representation of firing rates shows a tiny dynamic range. As we have previously anticipated, in this regime any given input in the first layer produces an approximate similar last layer output rate which is around the intersection between the *x* = *y* and the curve for the first layer (~60 Hz). As one increases weights by a little, *W_FF_* = 1.2 in Figure 2B, the input-output response and the gain for the first layer also increase generating a higher last layer output firing rate. Although the input-output response of the first layer is larger in slope and firing rate outputs of the FFN are higher, this behavior changes little in comparison to the previous case with a last layer showing a gain mostly flat which reflects a small dynamic range. These are input-output responses of the FFN when *J_AE_* ≤ *J_II_* which is similar to the transmission of firing rate through layers of Cond X in Figure 2B. As *J_AE_* increases, the input-output response of layers behaves even more non-linearly and despite the fact that between layer the transmission for low rates is 1:1, curves have a step function which saturates at inputs larger than 60 Hz (Cond Y in Figure 2D). Although this step profile is obtained at *W_FF_* = 1.2 (Figure 2B, Cond Y), this behavior is also seen for small *W_FF_*. Consequently, activity in higher layers ends either in a fixed firing rate close to the point in which the input-output curve of layer 1 crosses the *x* = *y* curve or saturates as the input-output slope is larger than 1. Therefore, if the transmission gain is less than or equal to 1 firing rates in higher layers go to an almost constant solution with small dynamic range whereas a gain larger than 1 produces increasing activity across layers which saturates. If the input-out slope of layer 1 never crosses the equality line, the firing rate propagation stays stable at low frequencies.

As it has been previously described, for small *W_FF_* a bump arises in which the firing rate outputs of layers become entirely independent of the initial input. We further study this bump by plotting the average output firing rate as a function of the feedforward layers for *W_FF_* of Figure 2C. As *W_FF_* = 0.6, the activity splits into two prime frequencies: one around 50 Hz, and the other approximately at the frequency of 20 Hz. The transmission at 10 Hz input produces a quick progression of rates to a high solution which then stabilizes at 70 Hz. That the firing rate is independent of the initial rate is explained because the feedforward chain develops strong spike correlations: neurons tend to synchronize with each other given the shared connectivity inputs. Given a small size network and a low gain of transmission a partial level of correlation from layer to layer is generated. By considering networks with more neurons, synchrony states would be avoided. Nonetheless, the system will still be constrained for the magnitude of the gain (see next section). A similar pattern of transmission has been previously described for a single (Brunel, 2000) or a chain of layers feedforwardly connected (Litvak et al., 2003). The exception here is that fluctuations coming from the reciprocal as well as the shared inputs are involved in the generation of correlated output spikes. Thus, the consequence of a small size network, and so, deviation of the balanced state, is an output entirely independent of the input firing rate which is important to avoid, if it desires, a linear transmission of firing rates.

### 3.3 Development of synchronous activity in the FFN

Firing rate transmission depends on both propagation of the mean rates and the variance of these rates through the network (Diesmann et al., 1999; van Rossum et al., 2002; Litvak et al., 2003). Given that neurons share common inputs, rates of different neurons produce correlations that merge activities of neurons into similar values. This creates that fluctuations of the population rates increase quickly across layers independent on the mean input rates. Here, we will explore again the problem of synchronous activity in a network with many layers. However, in our model, layers have internal feedback connections that could avoid correlations in the activity of neurons and therefore maintain low levels of fluctuation inside layers. As we have previously characterized, the gain can change the transmission modality of the FFN. Thus, we will analyze whether the gain smaller or larger than 1 produces propagation of synchronous activity in the chain. To achieve this, we observe the dynamics of the network by plotting the activity of 100 randomly chosen excitatory spike trains from 3 feedforward layers. Parameters are those indicated in Figure 2D, Cond W and Y.

The summary of these simulations is plotted in Figure 2F. It can be seen that the transmission of activity of the uncorrelated firing at constant *r*_0_ can result in two qualitatively different behavior in the deep layers. While in the regime with *J_AE_* < *J_II_*, case 1, activity propagates and is asynchronous across layer 1 to 10, for *J_AE_* > *J_II_*, case 2, spikes synchronize strongly and the activity saturates quickly as one moves to higher layers. Although the activity is asynchronous in early layers, a precise synchronous activity is produced as synfire waves traveling from layer to layer (not shown). These fluctuations increase through the network and develop an almost perfect correlation in layer 10. Despite the fact that we have only showed two representative cases of spike propagation, we have tested several other regimes but the issue of synchrony across feedforward layers always prevails as *J_AE_* > *J_II_*. Thus, considering these and previous findings, two general cases are observed: if the gain is smaller than 1, transmission of activity goes to a fixed rate and few correlations between spikes are observed whereas for gain larger than 1, correlations appear and modify the input-output response between layers. Fluctuations coming from the feedforward pathway as well as the internal feedback activity produce a net increase in correlation that is observed as synfire waves in the last layer. This is the pattern observed when *J_AE_* > *J_II_*. When *J_AE_* is larger, the rebound firing rate coming from inhibitory neurons increases the fluctuations inside layers which propagate and build up synchrony activity in the chain.

## 4. Feedforward network with a pulvinar structure

We now consider a FFN, our visual cortex, with an external structure attached, which mimics a higher order (HO) relay nucleus of the thalamus as the visual divisions of the Pulvinar (Pul) in primates (Sherman and Guillery, 2002). As previously described, a HO nucleus has two types of connections with the cortex: reciprocal (ubiquitous for all thalamic nuclei, arriving from cortical layer 6 and mostly feedback) and non-reciprocal (specific to HO, from cortical layer 5 and suggesting a strong feedforward component) (Sherman, 2007). For the purpose of this study we will only investigate the role of the thalamus as a parallel feedforward network rather than a reciprocal feedback with the cortex. Besides that, HO can indirectly link to neighboring cortical areas with long range interneurons (e.g., giant pulvinar inhibitory interneurons) that connect parts of the HO nucleus that are far apart (Imura and Rockland, 2006, 2007), allowing for an effective shortcut between distant cortical areas. Thus, we based the mean cortico-thalamic interaction of our model on specific neuroanatomical aspects.

More formally, *L* layers in the FFN are now connected to a pulvinar-like structure with *L* − 1 layers. Each of these layers is composed by excitatory (*E*) and inhibitory (*I*) neurons strongly connected. Connections between cortex and Pul have the following organization: excitatory (*E*) cortical neurons from layer ℓ = 1, …, *L*−1 project randomly to *E* and *I* neurons in sub-unit ℓ of the Pul, while *E* neurons in layer ℓ of the Pul project to *E* and *i* cortical neurons of layer ℓ + 1. Within the pulvinar, *E* neurons in layer ℓ = 1, …, *L*−1 project randomly to *E* and *I* neurons in layer ℓ + 1. These *i* neurons also have long range projections to *E* and *I* neurons in deeper pulvinar layers (For details see Section 2). Figure 1 indicates connections inside the model.

### 4.1 Logic of the cortico-thalamic-cortical model

We start by constraining the parameter of the cortico-pulvinar system to allow for solution in which the excitatory and inhibitory inputs are balanced. The consideration of balanced state in the networks might provide a linear input-output firing rate in the last cortical layer with weak spike correlations. Each layer of the cortico-pulvinar system was then settled with a strength of inhibitory recurrent connections larger than excitatory strengths. As we have described above, this will produce low firing rates in the FFN, if it is detached from Pul. But if the Pul is taken into account, the firing rates in the FFN may be higher, if the connections from the Pul are sufficiently strong, and the Pul is sufficiently active. In the Pul we must have *J^pul^_E0_*/*J^pul^_I0_* > *J^pul^_EI_*/*J^pul^_II_* > *J^pul^_EE_*/*J^pul^_IE_* and we further require that *J^PC^_EE_*/*J^PC^_IE_* > *J^pul^_EI_*/*J^pul^_II_*. We have also reduced the number of parameters and kept the balanced state by assuming that the recurrent connections are the same for both cortex and Pul. Consequently, *J^Ctx^_AE_* = *J^pul^_AE_* and *J^Ctx^_AI_* = *J^pul^_AI_* where *A* = *E, I*. For inhibitory long-range connections in the thalamus we assume *J^LR^_EI_* < *J^LR^_II_*. With such arrange the long-range inhibitory cells produce a final excitatory effective projection which results from the disinhibition of the inhibitory cells inside the Pul. This global inhibitory effect is stronger than the feedforward excitatory thalamic pathway. Finally we need to ensure that the input from the Pul cannot destroy the balanced state in the cortex which is guaranteed if (*J^CP^_EE_*/*J^CP^_IE_*) > *J^Ctx^_EI_*/*J^Ctx^_II_*. These minimal arranges in the cortico-pulvinar system should assure a nearly balanced state of both networks.

We further constraint the strength of connections by considering useful factors as *W_FF_*, factor that multiplies the strength of feedforward connections in the cortex. Similarly, we introduced a factor *W_PP_* which multiplies both *J^pul^_E0_* and *J^pul^_I0_* equally, the feedforward pulvinar strengths. Furthermore, we considered factors *W_PC_* and *W_CP_* which scale the strengths *J^PC^_AE_* and *J^CP^_AE_* respectively. Thus, from here on, we assumed that (*J^Ctx^_E0_*/*J^Ctx^_I0_*) = (*J^pul^_E0_*/*J^pul^_I0_*) = (*J^CP^_EE_*/*J^CP^_IE_*) =(*J^PC^_EE_*/*J^PC^_IE_*) = (12/7) and we use the factors *W_FF_, W_PP_, W_CP_*, and *W_PC_* to change the weights of these strengths. The nine different parameters were used to optimize the most linear input-output firing rate relation which also exploits the largest dynamic range of outputs (see Section 2). Table 1 shows effective values of connectivity for the strengths used in our model.

**Table 1:** **Parameter values for the cortex-thalamus network**.

**Parameter**	**Description**	**Value**
*J^Ctx^_E0_*	Feedforward or external connection strength from *E* to *E* cortical neurons	12
*J^Ctx^_I0_*	Feedforward or external connection strength from *E* to *I* cortical neurons	7
*J^pul^_E0_*	Feedforward connection strength from *E* to *E* pulvinar neurons	12
*J^pul^_I0_*	Feedforward connection strength from *E* to *I* pulvinar neurons	7
*J^PC^_EE_*	Connection strength from *E* cortical to *E* pulvinar neurons	12
*J^PC^_IE_*	Connection strength from *E* cortical to *I* pulvinar neurons	7
*J^CP^_EE_*	Connection strength from *E* pulvinar to *E* cortical neurons	12
*J^CP^_IE_*	Connection strength from *E* pulvinar to *I* cortical neurons	7
*J*^*Ctx*,*pul*^_*EI*_	Connection strength from *I* to *E* cortical or pulvinar neurons	21
*J*^*Ctx*,*pul*^_*II*_	Connection strength from *I* to *I* cortical or pulvinar neurons	15
*J^Ctx,pul^_AE_*	Connection strength from *I* to *A* cortical or pulvinar neurons	6
*J^LR^_EI_*	Pulvinar long-range connection strength from *I* to *E* neurons	13.934
*J^LR^_II_*	Pulvinar long-range connection strength from *I* to *I* neurons	19.45
*W_FF_*	Feedforward cortical factor that scales *J^Ctx^_E0_*/*J^Ctx^_I0_*	0.6
*W_PP_*	Feedforward pulvinar factor that scales *J^pul^_E0_*/*J^pul^_I0_*	0.15
*W_CP_*	Pulvino-cortical factor that scales *J^CP^_EE_*/*J^CP^_IE_*	2.0
*W_PC_*	Cortico-pulvinar factor that scales *J^PC^_EE_*/*J^PC^_IE_*	0.05234
*W_LR_*	Pulvinar long-range factor that scales *J^LR^_EI_*/*J^LR^_II_*	1
*K*	Average number of inputs	300
*N^Ctx^_E,I_*	Total number of *E* and *I* cortical neurons	3000
*N^pul^_E,I_*	Total number of *E* and *I* pulvinar neurons	600
τ^*E*^	E synaptic time constant	4 ms
τ^*I*^	I synaptic time constant	1 ms
τ*_m_*	Membrane time constant	10 ms
τ*_ref_*	Refractory period	5 ms

**A* can be excitatory (*E*) or inhibitory (*I*) populations*.

### 4.2 Transmission of activity in the cortico-thalamic-cortical loop

In previous work where the transmission of neuronal activity was simulated with rate model, parameter values which optimize for a linear firing rate transmission have shown that the strength of connectivity between cortex and the thalamus follows a pattern: connection strength from cortex to thalamus, *W_PC_*, has to be weak, whereas from thalamus to cortex, *W_CP_*, has to be strong. Furthermore, as it has been analytically demonstrated, the connectivity strength of the thalamic feedforward pathway, *W_PP_*, has to be much smaller in strength than the long-range connections, *J^LR^_EI_* and *J^LR^_II_* (Cortes and van Vreeswijk, 2012). A difference with the FFN, which is evident here, is that the new system needs low feedforward strength, *W_FF_*, to avoid synchrony across the two parallel chains. Taking these results as our starting point, we optimize under this regime the input-output curve of the last excitatory cortical population to obtain the most linear relationship and a dynamic range which exploits the whole range of frequency outputs. Thus, in the present model we found qualitatively similar parameter values to those from the rate based networks (see Table 1).

After optimizing the best cortical input-output firing rate curve we evaluate the transmission of information of the input rate throughout the cortico-pulvinar network by characterizing the firing rate output, synchrony and inactivation of the excitatory cortical neurons in layers. How the input propagates from one layer to the next was quantified by the average firing rate cortical outputs for inputs with rates between 10 and 100 Hz. Synchronous activity was estimated by measuring correlation between the spike counts of two joint spike count statistics (*n^i^*_1_ and *n^i^*_2_). After normalization of raw data in a binary sequence, pairwise correlation coefficient were determined by $\rho_{T}=(\text{Cov}(n_{1},n_{2}))/\sqrt{\text{Var}(n_{1})\text{Var}(n_{2})}$ where the correlation coefficient, ρ*_T_*, is a dimensionless quantity from −1 to 1 with the zero value denoting independent spiking neurons (de la Rocha et al., 2007). We also evaluate the percentage of inactivation of excitatory population of each layer when the firing rate is transmitted throughout the feedforward system. Because the activity of each layer is a function of the input average firing rate, the input-output relation of excitatory neurons is also investigated for both cortical and pulvinar layers. Thus, having these measurements, we explore in detail the achievements of the cortex-thalamus system.

**Figure 3:**
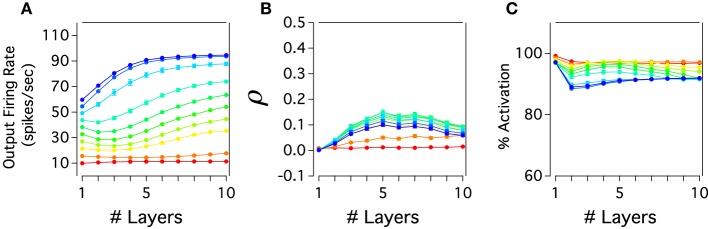
**Cortical propagation of activity in the cortex-thalamus network is mostly asynchronous and close to linear. (A)** The firing rate output of cortical layers is transmitted almost completely separately for each input rate applied in the cortex-thalamus network. Here, the firing rate of cortical layers for different input rates is plotted as a function of the increasing number of cortical layers. **(B)** The cortical correlation coefficient against number of cortical layers shows that the cortex develops in general low levels of synchrony activity. However, correlations tend to increase to later decrease as one moves from early to deep cortical layers. Furthermore, correlations are large at lower input rates than in higher ones. **(C)** Excitatory cortical neurons are mainly constantly activated through cortical layers: more than 90% of neurons in each cortical are activated. Color scale for average input rate similar to Figure 2B. Each curve is an average result of 20 independent simulations.

Figure 3 shows propagation, synchronization and percent of inactivation in cortical layers of the excitatory neurons. In Figure 3A, it can be seen that the firing rate propagation through cortical layer mainly increases as one move from layer 1 to 10. For low rate inputs, 10–15 Hz, the cortical activity is nearly constant since thalamic activity is neither evoked nor weakly activated when these inputs are applied to layer 1. On the contrary, higher firing rate inputs, 20–100 Hz, evoke thalamic activity of neurons which produces a monotonically increase of cortical activity gradually traveling across layers. This transmission is almost linearly maintained for each input applied to the system which results in a last cortical layer having separate firing rate outputs. Excitatory cortical layers also show a roughly constant activation. While low firing rate inputs produce constant cortical activation, this activation slightly decays in percent for higher inputs. For instances, around 90% of the total number of neurons in the system are activated and their activity remains constant throughout the cortical network (Figure 3B). If the activity is recorded over a longer period of time, all neurons will be active. Under these parameters of connectivity our model also shows weak synchronization between pairs of neurons with a ρ lower than 0.2 for every input injected. For 10–15 Hz inputs, when the thalamus is inactivated, the cortical activity travels almost asynchronously with ρ close to zero. For higher firing rates, ρ gradually increases and then decreases across layers having a maximum of correlation in layer 5 for 60–70 Hz inputs. Thus, excitatory cortical neurons of the cortico-pulvinar network are largely activated across layers having an asynchronous spike transmission with each input firing rate resulting in an almost different output rate.

**Figure 4:**
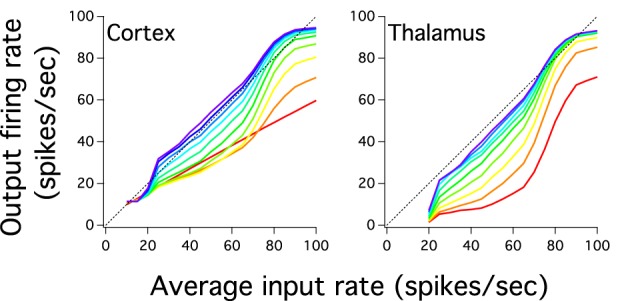
**The cortical and thalamic input-output response of layers shows that the cortex-thalamus system can transmit an input which varies gradually in magnitude**. Color scale for layers similar to Figure 2D. Input-output responses of networks show curve slopes gradually steeper and with a larger dynamic range as one goes to higher layers. Slope steepness and dynamic range are larger for layers in the thalamus than the cortex. However, firing rate outputs in the cortex are higher in deep layers than those seen in the thalamus. The last cortical input-output response shows a smooth close to linear curve.

The previous analysis we have done suggests that the excitatory cortical propagation has an input-output relationship which gradually increases more linearly and uses more of the dynamic range in deep cortical layers. We then explore whether the gain of this transmission, the slope of this curve, also reflects this progression of linearity by plotting the input-output relation of excitatory populations for both cortical and pulvinar layers (Figure 4). Except for the first cortical layer, which only receives input from the external source, input-output curves have a non-linear shape that increases in gain as one crosses the network to higher layers. For instances, for cortical layer 1 the gain is 0.55 and for layer 2, if the whole sigmoidal output is linearly fitted, the gain is close to 0.7. This tendency is also observed for the rest of cortical layers in which their gain gradually increases until layer 10 where the gain is close to 1 (Figure 4A). One can expect that the cortical firing rate output further increases if the number of layers expands. However, cortical layers 8, 9, and 10 have similar input-output curves which suggests a constant firing rate at least for the last three transmission steps. Nevertheless, for these three layers the network is partially saturated as higher cortical firing rates shows a constant frequency output (~94 Hz) even if the input gradually increases until 100 Hz. Pulvinar input-output curves have also sigmoidal shapes that increase in gain as one moves from layer 1 to 9 (Figure 4B). The difference with the case described above is that the thalamic gain is larger than those from cortical curves since thalamic curves are steeper. Another difference is the selective pulvinar spike response to certain firing rate inputs: low firing rate inputs (10 and 15 Hz) are too sparse to evoke thalamic activity which might be in accordance with electrophysiological pulvinar recordings (Wei et al., 2011). This particular non-thalamic activation in the last cortical layer shows an input-output curve with a large dynamic range between ~11.41 and ~94 Hz. For the last thalamic layer this interval is slightly larger, ranging from ~7 to ~93 Hz. We have also disconnected the Pul to the cortex to analyze spike propagation. As it is expected from Cond Z in Figure 2B, the low strength of cortical projections produces a poor representation of firing rate outputs with a last cortical layer having an almost single low frequency to any input applied into the network (not shown). Thus, we confirmed that the cortico-pulvinar network transmits almost linearly an input gradually varied in firing rate in which their input-output responses become partially steeper and exploit a large dynamic range as one moves from early to deep layers.

**Figure 5:**
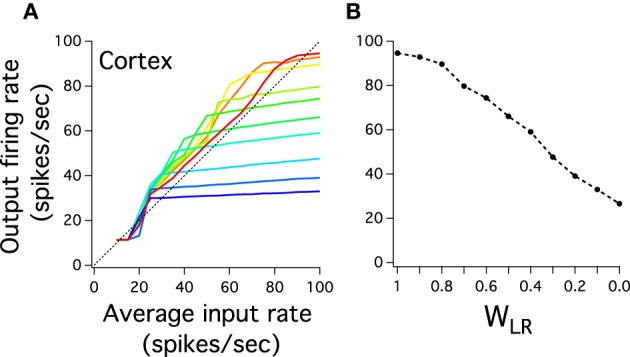
**Inhibitory long-range thalamic connections control the input-output response of the last cortical layer. (A)** The curve response of the last cortical decreases the dynamic range and increases the steepness as the strength of connection of the inhibitory neurons decreases. The red curve is the input-output last layer response previously described in Figure 5. Input-output curves decrease in *W_LR_* magnitude from red to blue curves. *W_LR_* multiplies equally *J^Th^_EI_* (*LR*) and *J^Th^_II_* (*LR*). The last blue color curve is when *W_LR_* = 0. **(B)** The highest value for each input-output cortical curves is plotted to show monotonic decrease as *W_LR_* goes gradually to 0. Each curve is an average result of 20 independent simulations.

A key assumption of our model is that pulvinar long inhibitory interneurons can indirectly link two distant cortical layers. This is the hypothesized shortcut property of the Pul to regulate cortical activity between low and high levels of its visual hierarchy. For instance, the large representation of firing rate in the last cortical layer maybe due to these long pulvinar interactions as it has been analytically shown in previous work (Cortes and van Vreeswijk, 2012). To test whether the input-output relation of the last cortical layer depends on variations of these interneurons we weight their strengths by a synaptic factor *W_LR_* to then gradually decrease them. The idea is to maintain the ratio *J^RL^_EI_*/*J^RL^_II_* constant but to reduce the effectiveness of these connections as *W_LR_* decreases. Figure 5A shows the best last cortical input-output relation (red curve) and input-output curves when *W_LR_* progressively decreases. The last cortical layer has two different behaviors: the input-output curves are gradually steeper and their dynamic range becomes smaller. All curves maintain the same low boundary output (11.41 Hz), but their maximum at 100 Hz input decreases monotonically as *W_LR_* decreases (Figure 5B). Cortical activity shows progressively a step curve as *W_LR_* approaches to zero where any high frequency input results in an approximately similar constant output. In fact, when *W_LR_* = 0 the last cortical curve shows a clear step response which is consequence of non-reciprocal connections between cortex and thalamus. The purely FFN does not have this sensitivity to firing rate inputs at these connection strengths (when *W_FF_* = 0.6, not shown). The long-range connections, thus, produce an effective increase of the cortical sensitivity: to a set of graded inputs the cortex can differentiate and smooth firing rate outputs given that the thalamus plays its role of a shortcut.

**Figure 6:**
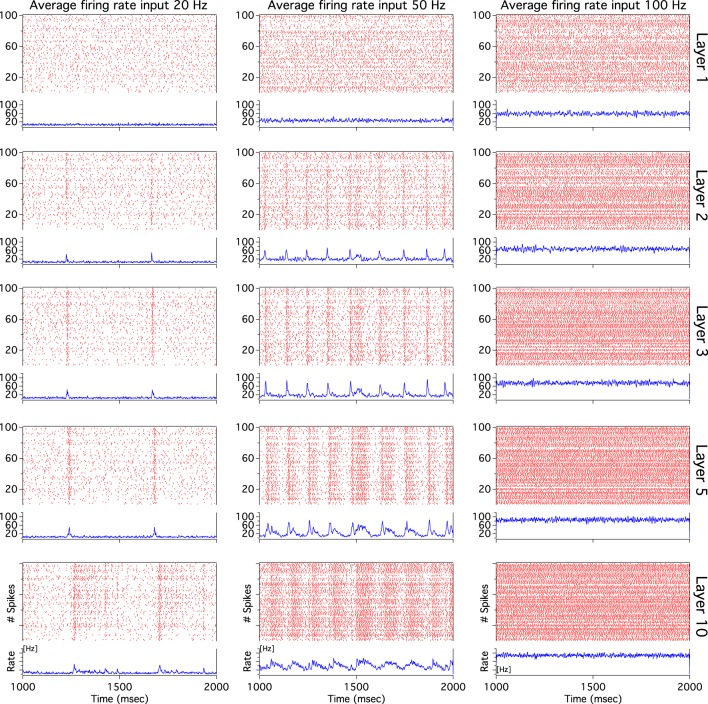
**The cortical activity in the cortex-thalamus system shows a bimodal solution**. Representative raster plots of 100 random chosen cortical neurons show that 20 and 50 Hz inputs generate an oscillatory low asynchronous activity in cortical layer 10. Similar raster plots for 100 Hz only present an asynchronous activity. Low oscillatory activity is observed with peristimulus time histogram (PSTH) of spikes. Low frequency oscillation is characterized for a “burst” activity of spikes in the rasters. Only excitatory neurons are shown

**Figure 7:**
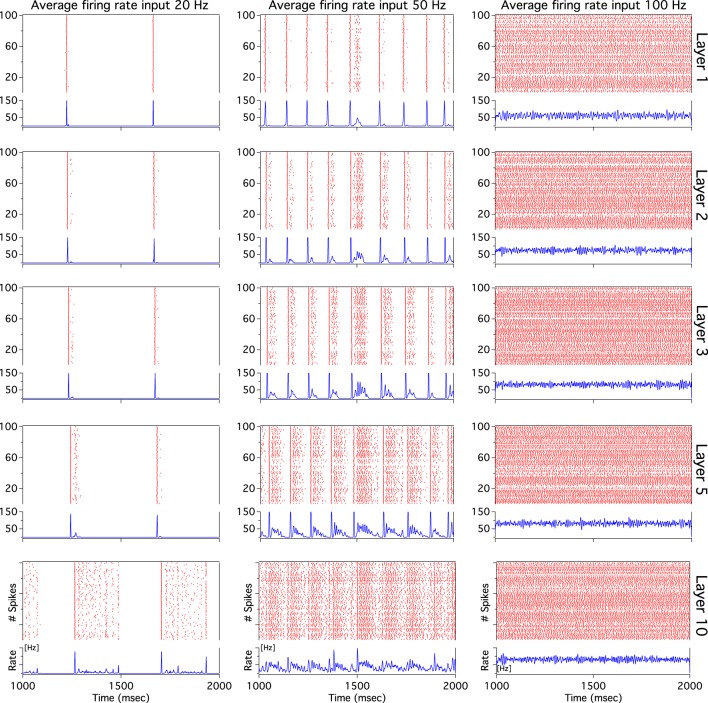
**The thalamic activity has similar bimodal activity to that of the cortex**. In the cortex-thalamus system, thalamic spikes have a strong synchronous burst activity with slow frequency observed at 20 and 50 Hz average inputs. PSTHs have a large peak at the beginning of the burst episodes which shows high correlation between thalamic spikes. At 100 Hz the representative raster plot of 100 excitatory random chosen spikes shows an asynchronous activity between units.

**Figure 8:**
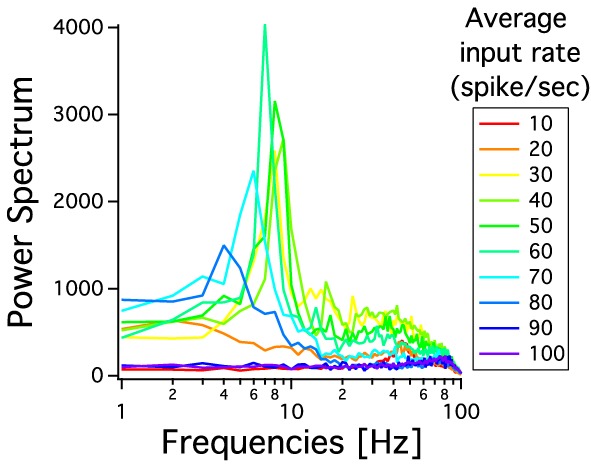
**Power spectrum of PSTHs from last cortical layer for different frequency inputs**. Each PSTH is previously low pass filtered at 100 Hz. The spectral analysis shows a predominant low oscillation between 2 and 11 Hz. The maximum periodicity is for a 60 Hz input. Each curve is an average result of 20 independent simulations.

As we have described before, low frequency rather than stronger inputs produced moderate correlation between excitatory spikes in the last cortical layer (Figure 3C). The cause of these correlations is neither the feedforward pathway alone nor because of the spikes incoming from the random input. To explore whether the Pul produces those low cortical correlations we plot 100 random chosen excitatory spikes from both cortical and pulvinar layer and their respective peristimulus time histograms (PSTH) were calculated as the cortico-pulvinar system receives three different frequency inputs (20, 50, and 100 Hz). Cortical and thalamic spikes show two types of transmission, one for low and another for higher frequency inputs (Figure 6). For inputs of 20 and 50 Hz, cortical layers show oscillatory synchronous volleys of “burst” spikes traveling across the network until layer 10 with a periodicity between 2 and 8 Hz (Figure 6). Cortical bursts are less conspicuous than those from the Pul where the latter has a sharp synchronous first spike followed by 4–7 irregular spikes (Figure 7). To an input of 100 Hz, however, both cortical and thalamic spikes are asynchronous and the periodicity in the PSTHs disappears (Figures 6, 7). For last layer we calculated then the power spectrum from the PSTH which measures the predominant frequency oscillation of spikes. While inputs in the range of 20–80 Hz generate high amplitudes of low PSTH oscillations, the rest of the tested frequencies has no strong impact inducing periodicities. Output oscillation ranged from 2 to 10 Hz with a maximum at 7 Hz for a 60 Hz input (Figure 8). Similar driving low oscillatory activity (alpha oscillations) from the Pul to cortical primate areas has been experimentally described when animals are under an attended selection task suggesting a control of the cortical transmission by the Pul (Saalmann et al., 2012). Thus, the cortico-pulvinar system produces a low oscillatory slightly synchronized firing rate if the network receives an input between 20 and 80 Hz or an irregular spiking output for the very both low (≤10 Hz) or high (≤90 Hz) spiking asynchronous input.

**Figure 9:**
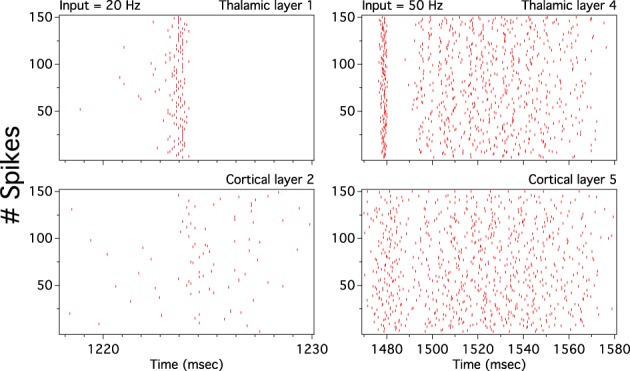
**Cortical spikes desynchronize the incoming synchrony burst activity from the thalamus**. A zoom on previous cortical and thalamic raster plots around a thalamic burst episode shows that cortical asynchronous spikes persist regardless of the strong synchrony activity from the thalamus. Remainder here that cortical layer ℓ receives input from both cortical area ℓ−1 and thalamic subdivision ℓ. Thus, uncorrelated cortical feedforward spikes wash out afferent synchronous thalamic bursts. Thalamic spikes generate an evoked pattern of cortical activity which is clearly observed as low oscillations in PSTHs of Figure 6.

Cortical low spike correlations and sharply synchronous thalamic bursts evoked at low frequency inputs maybe incoherent as global asynchronous transmission. Thalamic bursts propagate throughout the cortex where cortical bursts seem to have a predominant correlation in the first spike of the train than in the following units. Although at first sight it seems that cortical spikes copy the incoming thalamic burst, a more detailed observation inside cortical spike trains shows that units are asynchronous (Figure 9). Cortical output units are decorrelated even if one considers where the incoming burst is densest. The asynchronous cortical transmission is possible because the low cortical gain produces low cortical correlation which desynchronizes the incoming pulvinar bursts: the uncorrelated cortex washes out the thalamic synchrony. These and the previous findings show that the cortico-pulvinar network can transmit two types of information: the frequency of the firing rate input applied in the first cortical layer asynchronously and the slow burst oscillation from the thalamus at low frequency inputs.

**Figure 10:**
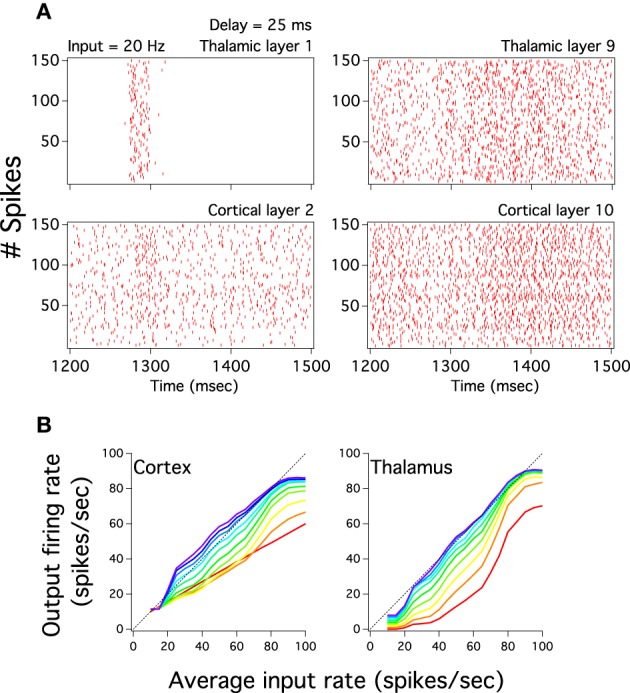
**Delayed pulvinar spikes still evoke close to linear cortical activity**. The cortical perturbation consists in pulvinar spike times randomly delayed in 25 ms. **(A)** Left and right columns plots show respectively the spike raster of pulvinar layer 1 and 9 (top) which projects to cortical layer 2 and 10 (bottom). Note that the current broad thalamic burst in layer 1 which partially projects at 1300 ms to cortical layer 2 vanishes in thalamic layer 9 and so in cortical layer 10. **(B)** Cortical and thalamic input-output response of layers for the perturbed cortico-pulvinar system. Color scale for layers similar to Figure 2D. The system still has a linear transmission of firing rate but both the dynamic range and the gain of last cortical input-output curves are smaller than those for the unperturbed system.

Given that pulvinar burst activates large both thalamic and cortical neural population we directly check whether it is imperative to have these pulvinar highly synchronized discharges to evoke cortical low frequency outputs. To that end, we decorrelated the spike trains from the Pul to the cortex by delaying this output with an homogeneous random time, ξ_τ_, inside the interval of (*t, t* + ξ_τ_ *D*), where *D* = 25 ms, so that the input from the Pulvinar to the cortex in Equation 2 is now characterized as *I*^*A*,ℓ^_*CP*,*i*_(*t*) = ∑_*j*_ *J*^*AE*,ℓ^_*ij*_(*CP*) *E*^*E*,ℓ−1^_*pul*,*j*_(*t* + ξ_τ_ *D*). While the delay desynchronizes pulvinar bursts at early cortical layers the periodicity, and so the burst, of these thalamic discharges vanish at deep both pulvinar and cortical layers even if the network is injected with a low input frequency (Figure 10A). We further explore whether the delay modifies transmission to inputs varied in frequency by plotting cortico-pulvinar input-output responses (Figure 10B). Although the propagation still remains approximately linear, the input-output responses show smaller both dynamic ranges and gains. In addition, curves do not increase as smoothly as in the unperturbed system (Figures 10B, 4). We have also exclusively delayed the spike time output of the inhibitory long-range interneurons but this fully stops the pulvinar burst discharges as well as the linear input-output transmission of the global network (not shown). Thus, even avoiding the incoming strong synchronized pulvinar burst to target cortical neuronal populations is possible to evoke low cortical firing rate and so asynchronously maintain an input graded in frequency until the last cortical layer.

## 5. Discussion

Several studies have suggested that the Pul of the thalamus regulates the cortical visual activity in primates. However, currently the thalamic role in processing information is unclear. In this paper we have tested, using a simplified FFN as a cortex, the implications of having a parallel Pul network connected to a visual cortex. We show that the Pul is necessary to transmit and maintain variation of an input rate through such a cortical chain, while preserving asynchronous transmission between layers. Thus, an almost linear input-output transmission between cortico-cortical (CC) areas is possible in a large sequential network as the visual cortex of primates. For the model we use here, the Pul provides oscillatory low frequency activity to the cortex which is transmitted across CC areas, if the input intensity is between 20 and 80 Hz. However, in the extended model in which we introduced random synaptic delay, we have found that these oscillations are not necessary to achieve faithful transmission of the input firing rate which is an advantage if another input targets the Pul, i.e., the superior colliculus (Kaas and Lyon, 2007). The implications observed in our model are a result of the restrictions we put on the network model.

Several computational studies have addressed the transmission of spiking activity through modular networks (Abeles, 1982; Aertsen et al., 1996; Diesmann et al., 1999; Litvak et al., 2003; Kumar et al., 2008, 2010; Goris et al., 2013; Jahnke et al., 2013). For instance, previous work has shown that the asynchronous activity can cross 6 layers of a feedforward chain if the strength of the connectivity, *W_FF_*, is strong (Vogels and Abbott, 2005). Although this analysis can be extended to a FFN of 10 layers (Figure 2), where an asynchronous rate transmission through chains is achieved, the last layer looses its linear input-output relation: firing rates converges into a single output regardless of the input frequency injected into the first layer. An alternative solution is to accept the closed functional connectivity between cortex and Pul. For a chain of 10 feedforward layers, the attached thalamus-like structure supplies the low gain of the cortical transmission without changing the strength of connections between both networks. This is highly convenient if one desires to quickly transmit a rate through layers because weights of connections do not have to be rearranged for each frequency input (Thorpe et al., 1996; Vanrullen and Thorpe, 2001). Furthermore, for a hypothetical network of more than 10 layers, it is a matter to adjust cortico-pulvinar weights to transmit asynchronous activity with varying firing rate. Thus, the cortico-pulvinar system partially solves the problem of an asynchronous transmission through a modular network and, as we will discuss, seems to also resemble experimental data.

## 6. Feedforward constraints

The transmission through a FFN produces a non-graded output activity in the last layer. In general, if the gain is less than 1, the input-output relationship of the last cortical layer is better described either by a constant value in low rates or a curve with positive slope but with a small dynamic range. These responses are also strongly dependent on the recurrent connections. If recurrent inhibitory connections are weak, spike correlation appears in the FFN. However, when the FFN has synchronous activity the input-output relation of the last cortical area is strongly non-linear and is characterized by a step function. Strong inhibitory weights avoid these correlation, but do not solve the problem of low gain. Thus, it is difficult to feedforwardly transmit realistic cortical activity because of the unrealistic output gain created through layers.

Although each layer of the FFN is in the balanced state, these results of feedforward transmission are similar to those in networks without recurrent connectivity inside layers. It has been suggested that excitatory and inhibitory inputs in each layer will avoid spike correlation if their connection strength satisfies the balance conditions (Litvak et al., 2003; Vogels and Abbott, 2005). While this result may be true for a large number of neurons, and so, a large number of connections between neurons (large *K*), it is not achieved for a limited population of neurons with finite number of connections. Although an asynchronous linear transmission is possible with our FFN, the input-output response of layers shows an unrealistic visual cortical transmission (Rolls and Baylis, 1986; Sclar et al., 1990; Avidan et al., 2002). The activity of neurons loose the gain from layer 1 and the firing rate tends to produce a non-graded output through layers. Even worse, an asynchronous activity transmission of the input is not always assured. Hence, a layered network with neurons in the balanced state neither can maintain and transmit linearly variation of an input rate nor solve the apparition of spike correlations between neurons when *K* is small.

This process is particularly recorded in the “bump” obtained when the *W_FF_* is weak. Here, output firing rates of layers vary non-linearly with the rate of the external input evoking pairwise spike correlations between neurons. Our interpretation is that the recurrent activity in each layer produces strong fluctuations which modify the frequency of the propagated input. Fluctuations coming from the feedback input will compete with fluctuations of the incoming input. Given that the feedforward input is weak, fluctuations of the recurrent input will persevere and generate spike correlation even in the case where the inhibition is larger in strength than the excitation (Figure 2C). Thus, the balanced state of neurons with a limited number of synapses may generate fluctuations which propagate at given values of lateral feedback connections. Another possibility is that the synaptic time of integration between neurons, τ*_syn_*, also influences this fluctuation observed in a feedforward transmission. The assumption of a possible propagation of fluctuation in a FFN with limited number of synapses per layer should be proved in future analysis.

## 7. Cortico-thalamic network

We have shown that a parallel shortcut structure added to a FFN transmits variation of an input gradually varying with rate which mimics the interaction between the visual cortical hierarchy and the Pul. The layered network propagates asynchronous activity between levels with a close to linear transmission. This propagation is qualitatively found when the strength of connectivity from cortex to thalamus is weak whereas the non-reciprocal input from thalamus to the cortex is strong. Furthermore, long-range connections have to be much larger than the local feedforward connections which reflect the thalamic shortcut attribute to create a graded asynchronous response in the last cortical layer. In fact, in the cortical-thalamic network, if the effective long-range connectivity is too weak the input-output response of the last cortical layer shows a much narrower dynamic range than when long-range connections are strong (Figure 5). Thus, as the simulations done in this study suggest, the Pul supplies the low transmission of the cortex increasing the effective gain and maintaining a linear propagation of the input. It is also important to emphasize that this hypothetical thalamic function can be extended to others HO nuclei (Llano and Sherman, 2008; Theyel et al., 2010).

In our model, there is a remarkable resemblance to experimental data with the burst activity found in our thalamic network. Recordings in monkey thalamus have shown that pulvinar neurons have a lower spontaneous burst profile of activity (Sherman, 2001; Wei et al., 2011). Although our thalamic network is constituted of very simple modeled neurons, the microcircuit arrangement of thalamic connectivity promotes the burst activity observed in our simulation. These bursts are a consequence of long-range inhibitory connections. Nevertheless, we are aware that the dynamics observed in our thalamic neurons is somewhat more complex than the simple connectivity of the thalamic network and model of neurons which have burst-like behaviors must be considered (Destexhe et al., 1998). More detailed theoretical work has to be done to better explain the dynamic of the thalamic burst activities and its incidence in the cortical activity.

A quite interesting result of our simulations lies in the fact that although thalamic burst activity is synchronous, the cortex shows low level of correlation between spikes. Previous theoretical literature has shown an inverse mechanism to obtain an asynchronous propagation (van Rossum et al., 2002). An uncorrelated activity in a FFN is transmitted approximately linearly as an external noise is applied. Although we believed *a priori* that for our cortico-thalamic network this noisy external source would be the thalamus it turned out to be the opposite. The cortical activity traveling across layers desynchronizes the correlated low frequency thalamic spikes because of the low cortical gain of transmission. Likewise, this combination of both the uncorrelated signal and the strong underground bursts produces a low cortical oscillation in the PSTHs. This oscillatory signal is only observed in a delimit frequency input range and may have some agreement with experimental data in which cortical activity is modified when the Pul is activated/suppressed (Soares et al., 2004; Logothetis et al., 2010; Theyel et al., 2010), as well as decreasing oscillations and synchrony activity of cortical cells (Molotchnikoff and Shumikhina, 1996), and presumably linking interconnected cortical areas in anesthetized and awake animals (Shumikhina and Molotchnikoff, 1999; Saalmann and Kastner, 2011; Saalmann et al., 2012).

Hence, as both experimental data and our results suggest, mixed activity can be propagated by the cortico-thalamic interaction. on one side the asynchronous activity of the feedforward pathway is transmitted linearly between layers, on the other, an oscillatory activity with slow frequency properties is provided by the thalamus. in our model which considers the propagation of activity through the cortico-thalamo-cortical loop, both signals can be transmitted without disturbing one another. however, to reproduce independently these two types of signals, thalamus and cortex have to be though as a single unit. if the thalamus is disconnected from the cortex, an asynchronous firing rate output activity is propagated through the cortex but the last cortical area has an unrealistic input-output response. otherwise, thalamic activation needs the cortex to evoke its response: experimental data shows that ho thalamic neurons have low spontaneous activity (Ramcharan et al., 2005), which turns in a high oscillatory output when animals are decorticated (Morison and Bassett, 1945). thus, about transmission of visual activity, the pul and the cortex should be viewed as single circuit and not as a sequence of parallel processing (Casanova, 2003).

### Conflict of interest statement

The authors declare that the research was conducted in the absence of any commercial or financial relationships that could be construed as a potential conflict of interest.
